# LIVER RESECTION FOR NON-ORIENTAL HEPATOLITHIASIS

**DOI:** 10.1590/0102-672020190001e1463

**Published:** 2019-12-20

**Authors:** Orlando Jorge Martins TORRES, Marcelo Moura LINHARES, Eduardo José B RAMOS, Paulo Cezar G AMARAL, Marcos BELOTTO, Angelica Maria LUCCHESE, Romerito Fonseca NEIVA, Theago Medeiros FREITAS, Rodolfo SANTANA, Josiel Paiva VIEIRA, Jaldo Santos FREIRE, Camila Cristina S TORRES, Antonio Nocchi KALIL

**Affiliations:** 1 Department of Gastrointestinal Surgery, Hepatopancreatobiliary Unit, Federal University of Maranhão, São Luís, MA, Brazil;; 2 Department of Gastrointestinal Surgery, Escola Paulista de Medicina, São Paulo, SP, Brazil;; 3 Department of Gastrointestinal Surgery, NS das Graças Hospital, Curitiba, PR, Brazil;; 4 Department of Gastrointestinal Surgery, Hospital São Raphael, Salvador, BA, Brazil;; 5 Department of Gastrointestinal Surgery, Santa Casa de São Paulo, SP, Brazil;; 6 Department of Gastrointestinal Surgery, Santa Casa de Porto Alegre, RS, Brazil.

**Keywords:** Hepatolithiasis, intrahepatic lithiasis, Non-oriental, Liver resection, Hepatectomy, Hepatolitíase, Litíase intra-hepática, Litíase intra-hepática não oriental, Ressecção hepática, Hepatectomia

## Abstract

**Background:**

Primary intrahepatic lithiasis is defined when the stones are formed in the liver and associated with local dilatation and biliary stricture. Liver resection is the ideal procedure.

**Aim:**

To evaluate the results of liver resection in the treatment of non-oriental intrahepatic lithiasis.

**Methods:**

Fifty-one patients with symptomatic benign non-oriental hepatolithiasis underwent surgical resection in six institutions in Brazil. Demography data, clinical symptoms, classification, diagnosis, management and postoperative course were analyzed.

**Results:**

Of the 51 patients, 28 were male (54.9%), with a mean age of 49.3 years. History of cholangitis was observed in 15 (29.4%). The types of intrahepatic lithiasis were type I in 39 (76.5%) and type IIb in 12 (23.5%), with additional type Ea in six (11.8%). Liver function test were normal in 42 patients (82.4%). Segmental atrophy was observed in 12 (23.5%). Treatments included left lateral sectionectomy in 24 (47.1%), left hepatectomy in 14 (27.5%) and right hepatectomy in eight (15.7%), with associated hepaticojejunostomy in four (7.8%). Laparoscopic liver resection was performed in eight (15.7%). Postoperative complications were observed in 20 (39.2%) with no mortality.

**Conclusion:**

Liver resection in patients with hepatolithiasis is the ideal procedure as it removes stones, stricture, atrophic parenchyma, and minimizes the risk of cholangiocarcinoma.

## RESUMO

**Racional:**

Litíase intra-hepática primária é definida quando os cálculos são formados dentro do fígado, podendo estar associada à dilatação local e estenosa da via biliar. A ressecção hepática é considerada o procedimento ideal.

**Objetivo:**

Avaliar os resultados da ressecção hepática no tratamento da litíase intra-hepática não oriental.

**Métodos:**

Cinquenta e um pacientes com hepatolitíase benigna não oriental sintomática foram submetidos à ressecção hepática em seis instituições no Brazil. Os dados demográficos, sintomas clínicos, classificação, diagnóstico, tratamento e evolução pós-operatória foram analisados.

**Resultados:**

Dos 51 pacientes havia 28 homens (54,9%), e a idade média era de 49,3 anos. História de colangite foi observada em 15 pacientes (29,4%). Os tipos de litíase observados foram tipo I em 39 (76,5%) e tipo IIb em 12 (23,5%), com o tipo adicional Ea em seis pacientes (11,8%). Os testes de função hepática estavam normais em 42 (82,4%) e atrofia segmentar foi observada em 12 (23,5%). O tratamento incluiu setorectomia lateral esquerda em 24 (47,1%), hepatectomia esquerda em 14 (27,5%) e hepatectomia direita em oito pacientes (15,7%). A hepaticojejunostomia esteve associada ao procedimento inicial em quatro (7,8%). Hepatectomia por videolaparoscopia foi realizada em oito (15,7%). Complicações pós-operatórias foram observadas em 20 pacientes (39,2%) e não houve mortalidade.

**Conclusão:**

Ressecção hepática na hepatolitíase é o procedimento ideal, pois remove os cálculos, a estenose, o parênquima atrofiado e minimiza os riscos para colangiocarcinoma.

## INTRODUCTION

Intrahepatic lithiasis is the presence of stones within the bile ducts, proximal to the right and left hepatic ducts, irrespective of the presence of gallstones in the gallbladder and/or common bile duct. It occurs more frequently in the 5^th^ and 6^th^ decades of life and there is no gender preference. It is a common disease in East Asia, which includes Japan, China, and South Korea, but rare in the West. Intrahepatic lithiasis is associated with complications such as biliary strictures, acute cholangitis, liver abscess, liver atrophy, secondary biliary cirrhosis, portal hypertension and hepatic failure. Furthermore, it is an important cause of intrahepatic cholangiocarcinoma^17^ ^,^ ^18^ ^,^ ^21^. 

Based on the causes of the disease, it is classified as primary or secondary. Primary intrahepatic lithiasis is defined when the stones are formed in the liver and associated with local dilatation and biliary stricture. The etiology has yet to be fully understood, although environmental, nutritional status, bile duct infection, cholestasis, parasites, the variation of bile duct, bile metabolic defect, and genetic factors are thought to contribute to the disease. In secondary intrahepatic lithiasis, stones are originally formed in the gallbladder or common bile duct and then migrate to the liver. Intrahepatic lithiasis is more common in the left lobe possibly because an acute angle between the common hepatic duct and the left hepatic duct which could induce bile stasis^2^ ^,^ ^5^ ^,^ ^13^ ^,^ ^17^. 

Clinical symptoms may include discomfort or epigastric abdominal pain, nausea, vomiting, jaundice and fever. Some patients are asymptomatic and the diagnosis is an incidental finding on abdominal imaging for unspecific symptoms. Migration of intrahepatic stones into the extrahepatic bile duct may cause pancreatitis as initial presentation^2^ ^,^ ^5^ ^,^ ^13^.

The objective of hepatolithiasis treatment is to remove the stones and ongoing infection, reduce the risk of recurrent stones, and prevent the risk of malignant transformation. Available treatments include medication, endoscopy and surgery^2^ ^,^ ^13^ ^,^ ^21^.

Liver resection, as well as biliary drainage, is commonly employed and represents the ideal procedure in the treatment of intrahepatic lithiasis because it can remove the damaged hepatic parenchyma, the stones, potential biliary stenoses and the diseased bile duct, avoiding the risk of subsequent cholangiocarcinoma. During the last decade, morbidity and mortality rates for liver resection have decreased significantly due to an increase of expertise of hepatopancreatobiliary surgeons and intensive care unit^2^ ^-^ ^5^ ^,^ ^13^ ^,^ ^21^. 

The aim of this study was to evaluate the results of liver resection as treatment of non-oriental intrahepatic lithiasis. 

## METHODS

Ethical approval was not required and patient identifying knowledge was not presented in this report. Between March 2010 and June 2018, 51 patients with symptomatic benign non-oriental hepatolithiasis underwent surgical resection in six institutions in Brazil. Data related to age, gender, clinical symptoms, location of the stones, intraoperative diagnosis, and postoperative course are presented.

The diagnosis, extent and severity of the intrahepatic lithiasis were evaluated in all patients using liver function tests and imaging including abdominal computed tomography scan, magnetic resonance imaging or magnetic resonance cholangiopancreatography (MRCP) in all patients. 

The lithiasis was typed according to Dong’s classification^2^. In type I, the stone is localized, unilobar or bilobar. The type II has diffuse stone disease, without atrophy of the hepatic parenchyma or stricture of the intrahepatic bile ducts (IIa), segmental atrophy or/and stricture of the intrahepatic bile ducts (IIb), biliary cirrhosis and portal hypertension (IIc). The presence of extrahepatic stones is classified as type E, with normal sphincter of Oddi (Ea), relaxation of the sphincter of Oddi (Eb), or stricture of the sphincter of Oddi (Ec)^2^.

In order to identify the possibility of cholangiocarcinoma the clinical symptoms, MRCP and the tumor marker CA 19-9 (over 200 U/ml) were utilized. Liver resection was indicated in symptomatic patients due to the presence of stones leading to cholangitic abscesses, biliary stenosis, fibrosis or atrophy of the liver. Intraoperative cholangiography and Roux-en-Y hepaticojejunostomy was performed in selected cases according to surgeon discretion. Histopathological evaluation of the resected liver to identify cholangiocarcinoma was performed routinely and the disease was staged. The patients were followed up by clinical evaluation, laboratory data and MRCP.

Residual stones were defined as calculi within the intrahepatic ducts within three months after resection and recurrent stones calculi detected afterwards^2^ ^,^ ^13^. 

## RESULTS

A total of 51 patients underwent liver resection for intrahepatic lithiasis during the study period. Among these, 28 (54.9%) were male and 23 (45.1%) female, with a mean age of 49.3 years (26-78). History of cholangitis was observed in 15 patients (29.4 %), isolated abdominal pain in 13 (25.5%), fever and abdominal pain in 11 (21.5%), jaundice and pain in five patients (9.8%, Table 1).

**TABLE 1:** Clinical presentation of the patients

Clinical presentation	n	%
Cholangitis	15	29.4
Abdominal pain	13	25.4
Fever and abdominal pain	11	21.5
Jaundice and pain	5	9.8
Weight loss	5	9.8

Previous biliary procedures were observed in 32 (62.7%), which include laparoscopic or open cholecystectomy with or without common bile duct exploration in 28 patients (54.9%), hepaticojejunostomy in 13 (25.5%) and endoscopic retrograde cholangiopancreatography in four (7.8%). In nine (17.6%) the etiology was identified, including patients with biliary stricture after laparoscopic cholecystectomy in five (9.8%). 

Liver function test were normal in 42 patients (82.4%). Child-Pugh class B was observed in nine (17.6%). Serum bilirubin levels were analyzed and were increased in nine (17.6%) patients, and alkaline phosphatase and gamma-glutamyl transpeptidase were increased in 18 (35.3%) and 28 (54.9%), respectively. Other liver function tests were within normal limits. Leukocytosis was observed in eight (15.7%) patients. Assessment for tumor marker CA 19-9 was performed in nine (17.6%), and was elevated in two (22.2%). 

Preoperative diagnostic evaluation included ultrasonography in 14 (27.5%) patients, magnetic resonance with MRCP in 46 (90.2%), and computed tomography scan in 21 (41.2%). Diagnostic and therapeutic endoscopic retrograde cholangiopancreatography had been performed in two (3.9 %) patients after MRCP. The procedure included papillotomy and biliary stenting to relief biliary sepsis in acute cholangitis. 

**FIGURE 1:**
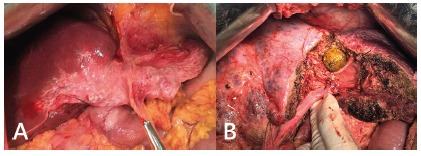
A) Segmental atrophy in patient with hepatolithiasis; B) huge intrahepatic stone

**FIGURE 2:**
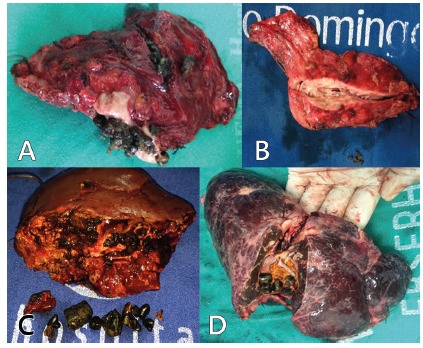
A and B) Left lateral sectionectomy due to hepatolithiasis in patient with atrophy; C and D) without

The types of intrahepatic lithiasis based on Dong classification were type I in 39 (76.5%) and type IIb in 12 (23.5%, Figure 2). Additional type Ea (extrahepatic stones in type I) was observed in six (11.8%). Segmental liver parenchyma atrophy was observed in 12 (23.5%, Figure 1)^3^.

**FIGURE 3:**
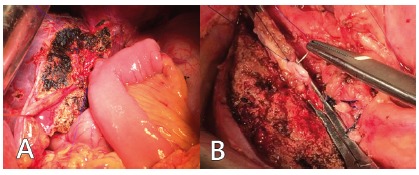
A) Hepaticojejunostomy after left lateral sectionectomy; B) intrahepatic bile duct is occluded after clearance

Surgical treatments included left lateral sectionectomy in 24 (47.1%, Figure 2), left hepatectomy in 14 (27.5%) and right hepatectomy in eight (15.7%, Table 2). Associated hepaticojejunostomy was performed in four (7.8%, Figure 3A) as a result of grossly dilated intrahepatic duct. All surgeries included bile duct exploration, but common bile duct stones were observed in 19 patients (37.2%). T-tube drainage procedure was not performed in this study. Six (11.8%) underwent liver resection due to associated liver abscess. Laparoscopic liver resection was performed in eight (15.7%) who underwent left lateral sectionectomy. The mean duration of operation was 230 min (190-300). 

**TABLE 2:** Surgical treatment of hepatolithiasis

Surgical procedure	n	%
Left lateral sectionectomy	24	47.1
Left hepatectomy	14	27.5
Right hepatectomy	8	15.7
Right posterior sectionectomy	3	5.9
Bisegmentectomy 5-6	1	1.9
Non-anatomic hepatectomy	1	1.9
Additional hepaticojejunostomy	4	7.8

Biliary stone clearance associated with liver resection was performed in 37 patients (72.5%) intraoperatively through intrahepatic dilated bile duct. After clearance, the intrahepatic bile duct was closed (Figure 3B). Postoperative complications were observed in 20 (39.2%) patients and included surgical site infection, biliary fistula and pneumonia (Table 3). The frequency of infectious complications was relatively more after bile enteric anastomosis; surgical site infection being the main complication observed. 

**TABLE 3:** Complications

Postoperative complications	n	%
Surgical site infection	9	17.6
Pneumonia	4	7.8
Biliary fistula	4	7.8
Pleural effusion	2	3.9
Bleeding	2	3.9
Acute renal failure	2	3.9
Subphrenic abscess	1	1.9

Forty-two patients (82.3%) were followed-up and nine (17.6%) were lost. The follow-up range from 6 to 38 months with a median time period of 25 months. No mortality was observed during follow-up. The results were defined as good when there were no complications related to the procedure or due to recurrence of stones as liver abscess or cholangitis during the follow-up period. In this study no cholangiocarcinoma was diagnosed preoperatively but an intra-hepatic cholangiocarcinoma was observed in a single case as histopathological finding of the specimen (1.9%). The patient underwent chemotherapy. Residual stones were observed in an asymptomatic patient after routine evaluation, and the patient was followed-up without intervention. Stone recurrence was observed in two asymptomatic patients (3.9%) and without additional procedure. 

## DISCUSSION

Intrahepatic lithiasis is endemic to East Asia with prevalence that range from 30% to 50% of patients undergoing surgery for cholelithiasis. The disease is not common in the western world, with a prevalence of 0.6-1.3% and different etiologies have been observed. The characteristics of the intrahepatic stones treated in Brazil are similar to those with oriental hepatolithiasis. We applied the treatment protocol according to the presence of symptoms and the location of the disease, with liver resection for unilateral disease. To our knowledge, this study represents the largest Brazilian series of liver resection for hepatolithiasis^2^ ^,^ ^5^ ^,^ ^6^. 

The management of intrahepatic lithiasis requires to remove all the stones completely, the strictured ducts if present and promote adequate drainage of the affected segment of the bile ducts to the small intestine. Liver resection is the only treatment which reduces the risk of recurrence and aggressive procedure is necessary to avoid complications such as secondary biliary cirrhosis, suppurative cholangitis, portal hypertension and liver failure. The recurrence rate of intrahepatic lithiasis following liver resection is significantly lower when compared with choledocholithotomy or biliary-enteric anastomosis (hepaticojejunostomy) alone or nonsurgical approaches. Hepatectomy is relatively safe in the treatment of unilateral hepatolithiasis and the rate of clearance of hepatolithiasis is high with a small amount of residual stones. In patients with biliary stricture, percutaneous approach is better than endoscopic management, providing temporary relief, but in case of failure or recurrence, surgical treatment is indicated. In this work liver resection was indicated in symptomatic patients with complications as liver abscess, stenosis or segmental liver atrophy, in patients with irreversible disease^7^ ^,^ ^8^ ^,^ ^16^ ^,^ ^11^.

After resection some authors drained the common bile duct with a T-tube. This procedure is related to some complications such as bile leak and biliary tract infection. In addition some patients must carry the T-tube for weeks, which cause discomfort. We did not use T-tube.^6^ ^,^ ^7^ ^,^ ^11^ ^,^ ^16^

Residual stone was observed in a single patient (1.9%) and recurrence rate stone was 3.9% (n=2), a lower rate when compare with others studies. The residual stone rate is 20-35% in the majority of studies without liver resection. Better results are observed when the surgery is planned. A single surgical procedure and avoiding repeated manipulation of biliary tract is necessary to achieve optimal outcome. The atrophied liver should be resected and the bile duct explored to avoid residual stone^5^ ^,^ ^7^ ^,^ ^9^ ^,^ ^16^ ^,^ ^18^. 

Bilioenteric anastomosis was indicated when the bile duct in the liver surface was dilated of 2 cm or more and hepaticojejunostomy was performed in all patients, as in the majority of studies. Other procedures like cutaneous hepaticojejunostomy were not employed. Hepaticojejunostomy is associated with complications such as recurrence of symptoms, cholangitis and liver abscess. Different techniques as choledochojejunostomy and choledochoduodenostomy are associated with biliary reflux and gastrointestinal dysfunction. In case of stones associated with significant dilation and biliary drainage procedure was required, the success rate decreases to 80%. In this series four patients underwent additional hepaticojejunostomy and biliary fistula was observed in one case. Hepaticojejunostomy was associated with worse results than patients without extrahepatic dilations, probably due to presence of extrahepatic biliary disease, stone formation and inadequate biliary drainage. Biliary reflux and gastrointestinal dysfunction are common after choledochojejunostomy and hepaticojejunostomy. Recurrence of symptoms showed that these procedures are not the ideal solution for intrahepatic lithiasis. However, a better evaluation of the therapeutic impact of liver resection and biliary tract exploration with and without biliary drainage on intrahepatic lithiasis is necessary^1^ ^,^ ^6^ ^,^ ^8^ ^,^ ^9^ ^,^ ^15^ ^,^ ^16^. 

Liver resection for the treatment of patients with intrahepatic lithiasis has been reported to be associated with a low rate of recurrent cholangitis, liver abscess or recurrence stones. Better late results are achieved in patients with unilateral stones who did not present extrahepatic biliary disease. In these cases all the compromised liver parenchyma can be removed, and the patient can be cured^6^ ^,^ ^7^ ^,^ ^11^ ^,^ ^13^ ^,^ ^16^. This study showed that liver resection is safe and effective for the treatment of intrahepatic lithiasis when indicated for unilateral disease with biliary stenosis, parenchyma fibrosis or atrophy. For unilateral intrahepatic lithiasis, liver resection has low morbidity and mortality, with better success rate when compared with endoscopic or percutaneous approach^6^ ^,^ ^7^. 

The incidence of complications such as biliary fistula to the wound surface should not be high. The wound surface of the liver should be aligned as much as possible to avoid the occurrence of bile leak. The rate of major complications here is relatively low. There was no operative mortality even in patients with cholangitis or liver abscess^1^ ^,^ ^8^ ^,^ ^10^ ^,^ ^11^ ^,^ ^19^ ^,^ ^20^. 

Cholangiocarcinoma associated with intrahepatic lithiasis is reported in the literature ranging from 1.5-18%. In this work cholangiocarcinoma was not identified preoperatively and just one patient presented with cholangiocarcinoma after pathological evaluation. Herman et al^5^ suggested that chronically inflamed liver tissue could play some role in the development of cancer. Liver resection could avoid the evolution to cholangiocarcinoma^1^ ^,^ ^5^ ^,^ ^6^ ^,^ ^15^. 

When hepatolithiasis involved two lobes the treatment is complicated and relatively more difficult. Two segments resected in each lobe are necessary in some cases. Extensive liver resection is not recommended for patients with intrahepatic lithiasis, especially those without disease of the left medial sector. In one case the liver was opened to access a huge stone, resulting in more intra-operative bleeding and biliary fistula^9^ ^,^ ^12^ ^,^ ^14^ ^,^ ^15^ ^,^ ^19^ ^,^ ^20^.

Open hepatectomy is the standard approach to perform liver resection but laparoscopic left lateral segmentectomy is considered a routine procedure for experienced hepatobiliary surgeons. Laparoscopic liver resection has showed significantly shorter mean postoperative hospital stay without difference in the rate of complications. Deformed biliary anatomy and perihepatic adhesions in patients with intrahepatic lithiasis may increase the risk of complications. The mean operation time was significantly shorter for laparoscopic group than for the open group as has been observed by other authors. Laparoscopic hepatectomy is safe and effective for well-selected patients^17^ ^-^ ^19^.^^

## CONCLUSION

Liver resection in patients with hepatolithiasis is considered the ideal procedure as it removes stricture, the atrophic parenchyma, and the stones and minimizes the risk of cholangiocarcinoma. 
